# Disseminated *Mycobacterium marinum* presenting as bursitis in a patient with psoriatic arthritis on adalimumab

**DOI:** 10.1016/j.jdcr.2025.10.020

**Published:** 2025-10-28

**Authors:** Mallory Suhling, Aditya Dutt, Olivia Cook, Summer Moon, Sheva Khalafbeigi

**Affiliations:** a HCA Healthcare/USF Morsani College of Medicine GME: HCA Florida Largo Hospital, Largo, Florida; b Midwestern University Chicago College of Osteopathic Medicine, Downers Grove, Illinois

**Keywords:** adalimumab, bursitis, disseminated *Mycobacterium marinum*, fish tank granuloma, Florida, *M. marinum*, *Mycobacterium marinum*, psoriatic arthritis, TNF, tumor necrosis factor

## Introduction

*Mycobacterium marinum* is a nontuberculous, slow-growing, acid-fast bacillus (AFB) found in aquatic environments.^1^^,^^2^ Risk of nontuberculous mycobacteria is well documented in patients on tumor necrosis factor (TNF)-α inhibitors.^3^ Adalimumab, a human monoclonal antibody targeting TNF-α, is approved for treatment of multiple inflammatory diseases, including psoriatic arthritis.^3^ TNF-α plays an integral role in granuloma formation and host defense against mycobacteria.^2^^,^^3^ Although cases of *M. marinum* in patients on adalimumab for psoriasis have been reported, only 3 involve psoriatic arthritis, and all involve limited cutaneous disease.^3^, ^4^, ^5^ We report a case of disseminated *M. marinum* infection in a psoriatic arthritis patient on adalimumab, presenting initially as olecranon bursitis.

## Case report

A 62-year-old male with psoriatic arthritis on adalimumab 40 mg subcutaneous injection every 2 weeks and prednisone 5 mg daily developed a pustule on the left olecranon which evolved into a painful 2 cm nodule over 3 months. He recalled recent snorkeling in the Florida Keys and frequent yard work. Examination revealed a soft, mobile subcutaneous mass with overlying erythema. Ultrasound and magnetic resonance imaging revealed nonspecific subcutaneous edema without bony involvement.

Orthopedics diagnosed bursitis and performed multiple drainages and debridements over 6 months with no relief. Culture performed in specialized labs eventually revealed *M. marinum*. Adalimumab was discontinued, and antibiotics were initiated–initially doxycycline and trimethoprim/sulfamethoxazole, then switched to clarithromycin 500 mg orally twice daily, rifampin 300 mg orally twice daily, and ethambutol 1600 mg orally once daily by infectious disease. Clarithromycin was later replaced by azithromycin 500 mg orally daily.

**Fig 1:**
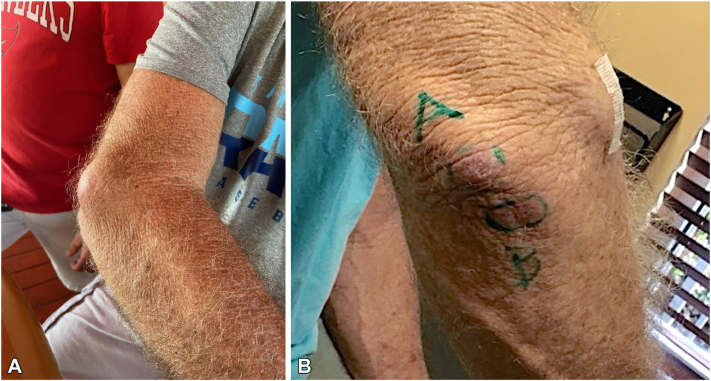
Right elbow. **A,** A painful, enlarging subcutaneous mass developed in the setting of recurrent left elbow bursitis and history of recent snorkeling. **B,** New cutaneous lesions developed after debridement.

**Fig 2:**
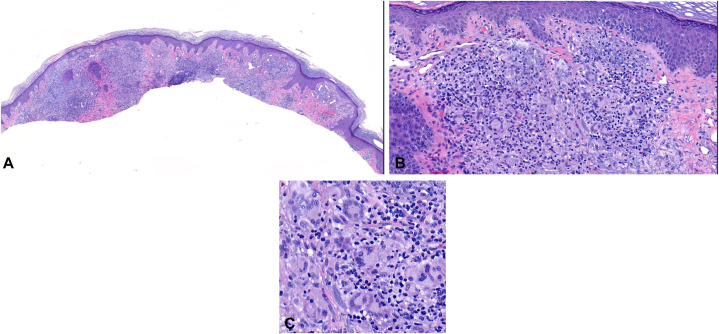
Histopathology of the right elbow. **A,** Suppurative and granulomatous dermal infiltrate. H&E, magnification, scanning power **(B)** Foreign body giant cell reaction comprising inflammatory nodular infiltrate. H&E, magnification, 40× **(C)** Lymphohistiocytic infiltrate with numerous multinucleated giant cells. H&E, magnification, 100×.

Despite treatment, a new tender, erythematous papule developed on his right elbow, and the original lesion persisted (Fig 1). Biopsy of the new lesion revealed suppurative and granulomatous inflammation and foreign body giant cell reaction but negative acid-fast bacillus, Fite, and fungal stains (Fig 2). He provided supplementary history, recalling a 30-pound unintentional weight loss over 3 and a half months, fatigue, and a positive tuberculosis test coincident with his first lesion. A follow-up chest radiograph ordered by his primary care practitioner showed no evidence of tuberculosis or other pulmonary pathology. Bilateral elbow debridement followed, and cultures were again AFB positive. Blood cultures were negative for *Mycobacteria*, and basic laboratory studies, including a complete blood count and comprehensive metabolic panel, were unrevealing.

**Fig 3:**
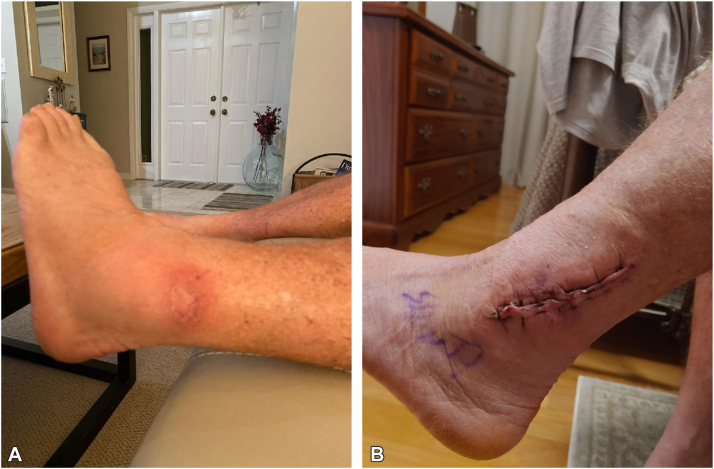
Left ankle. **A,** Despite antibiotic therapy, a new painful mass developed. **B,** Ankle healing well following debridement and washout.

After 5 months of antibiotic therapy, the elbow lesions resolved. Shortly after, a large, painful, draining left ankle mass developed, requiring debridement (Fig 3). Radiograph demonstrated edema without bony change, and AFB was negative. He remained on azithromycin and ethambutol, with trimethoprim/sulfamethoxazole 100 mg orally twice daily replacing rifampin due to vertigo, for several additional months following the procedure. At the time of writing this, no new lesions have appeared in 5 months, and he completed a 10-month course of antibiotics. His joint pain has returned since discontinuation of adalimumab, necessitating a short prednisone taper as well as diclofenac 75 mg orally twice daily.

## Discussion

*M. marinum* was first identified in fish in 1926 and later linked to human infections from pools and aquariums.^1^^,^^2^^,^^6^^,^^7^ Diagnosis is often delayed–on average, by 12 months–due to subjectively insignificant inoculating injuries and long incubation periods of 2 to 8 weeks, sometimes up to 9 months.^2^^,^^3^^,^^6^^,^^7^ Cutaneous lesions develop slowly and painlessly, potentially delaying presentation.^2^^,^^6^^,^^7^ Our patient reported fatigue and weight loss, subtle and nonspecific symptoms, which can be overlooked.^2^^,^^6^ Most cases remain localized to skin and soft tissue, presenting as a solitary papular, nodular, or plaque-like lesion.^1^^,^^2^^,^^6^^,^^7^ Locally invasive and disseminated disease occurs rarely and primarily in immunosuppressed individuals.^1^^,^^2^^,^^6^ Low-dose prednisone may have contributed to mild immunosuppression and disseminated infection in our patient.

Diagnosis is challenging due to nonspecific histology and frequent false negativity of AFB and Fite stains.^1^^,^^6^^,^^7^ Noncaseating or necrotizing granulomas, lymphohistiocytic dermal infiltration, and giant cells are present on microscopy in most cases, though, these features are not pathognomonic.^1^^,^^2^ Many labs are not equipped to culture *M. marinum*, which grows best at 30-32 °C and is inhibited at 37 °C.^1^^,^^2^^,^^6^ Cultures must be monitored for at least 6 weeks, further prolonging diagnosis.^3^ New molecular techniques are faster, but high homology between *M. marinum* and *M. ulcerans* poses a challenge.^1^^,^^2^^,^^6^^,^^7^ Many cases will have a positive tuberculin skin test and interferon-gamma release assay due to cross-reactivity with *M. tuberculosis*.^1^^,^^2^^,^^6^^,^^7^

Workup for disseminated *M. marinum* is not clearly defined. Recommended evaluations include imaging of symptomatic musculoskeletal sites to assess for joint or bone involvement, chest imaging to exclude pulmonary disease, and routine laboratory studies. Blood cultures are usually unrevealing since *M. marinum* does not grow well at 37 °C. Our patient underwent chest radiography, imaging of involved extremities, and basic laboratory workup, which confirmed disseminated disease was confined to the skin and joints without visceral involvement.

No standardized treatment exists.^1^^,^^2^^,^^7^ For superficial infections, tetracycline monotherapy may suffice, but multi-drug regimens (eg, ethambutol and clarithromycin) are preferred.^1^^,^^3^^,^^6^^,^^7^ Rifampin is added for bone or joint involvement.^1^, ^2^, ^3^^,^^6^ Treatment should be continued for 1 to 2 months after complete resolution of all lesions.^1^, ^2^, ^3^^,^^7^ Surgical debridement remains controversial but is often utilized for aggressive or refractory disease.^1^^,^^2^^,^^6^^,^^7^

TNF-α inhibitors impair granuloma formation and cell-mediated immunity, which can lead to atypical mycobacterial infections.^2^^,^^3^ For serious infections, guidelines recommend TNF-α inhibitor discontinuation, antibiotic initiation, and low-dose corticosteroids or NSAIDs for psoriatic arthritis management. There is no consensus regarding psoriatic arthritis treatment following resolution of a nontuberculous mycobacteria infection, creating a clinical challenge for psoriatic arthritis management post-infection. However, non-TNF agents with lower infection risk, such as apremilast, IL-17 or IL-23 inhibitors, or abatacept, are advised.^8^^,^^9^

*M. marinum* is ubiquitous and can survive over 2 years in soil and water.^6^ With rising aquarium use and increased water exposure due to flooding in regions like the southeastern U.S., incidence is expected to increase.^6^^,^^7^

## Conflicts of interest

None disclosed.
